# Supplementary material for: An autoregulatory cell cycle timer integrates growth and specification in chick wing digit development
Source: eLife. 2019 Sep 23;8:e47625. doi: 10.7554/eLife.47625 (PMC6777937; doi:10.7554/eLife.47625)
Supplement: Figure 4—source data 1. — (A) DMEM 6 h. (B) PD0332991 6 h. (C) DMEM 24 h. (D) PD0332991 24 h. (E) DMEM 6 h. (F) Cyc 6 h. (G) PD0332991 6 h. (H) Cyc/PD0332991 6 h. (I) DMEM 24 h. (J) 10 Cyc 24 h. (K) PD0332991 24 h. (L) Cyc/PD0332991 24 h. [file elife-47625-fig4-data1.docx]

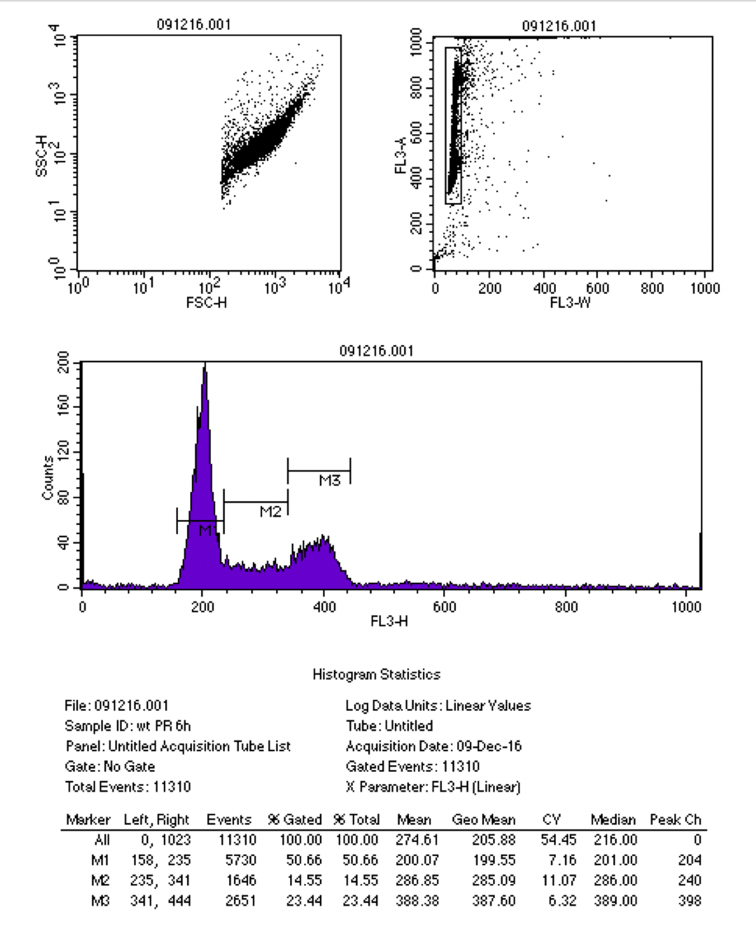


Figure 4—source data 1A. DMEM 6h­


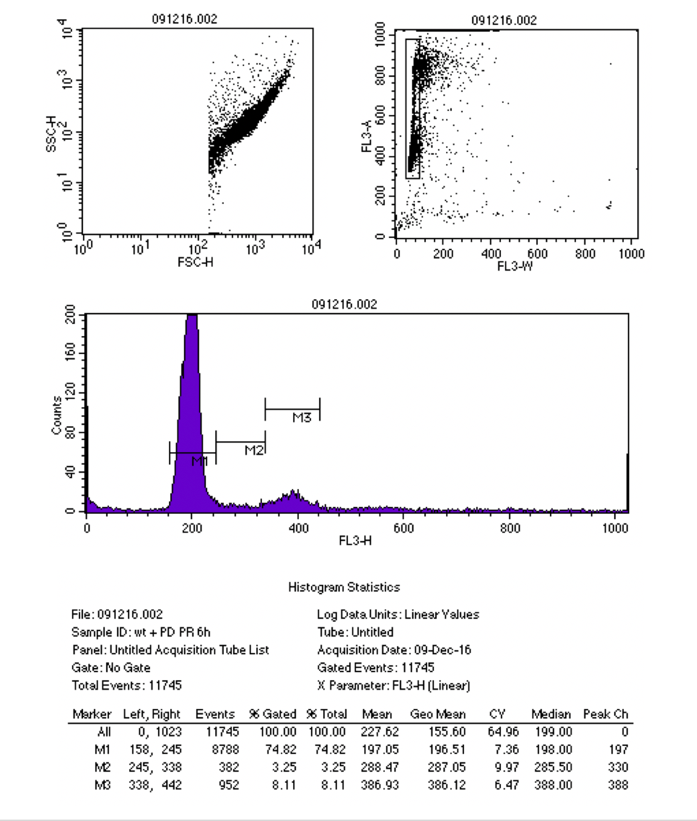


Figure 4—source data 1B. PD0332991 6h


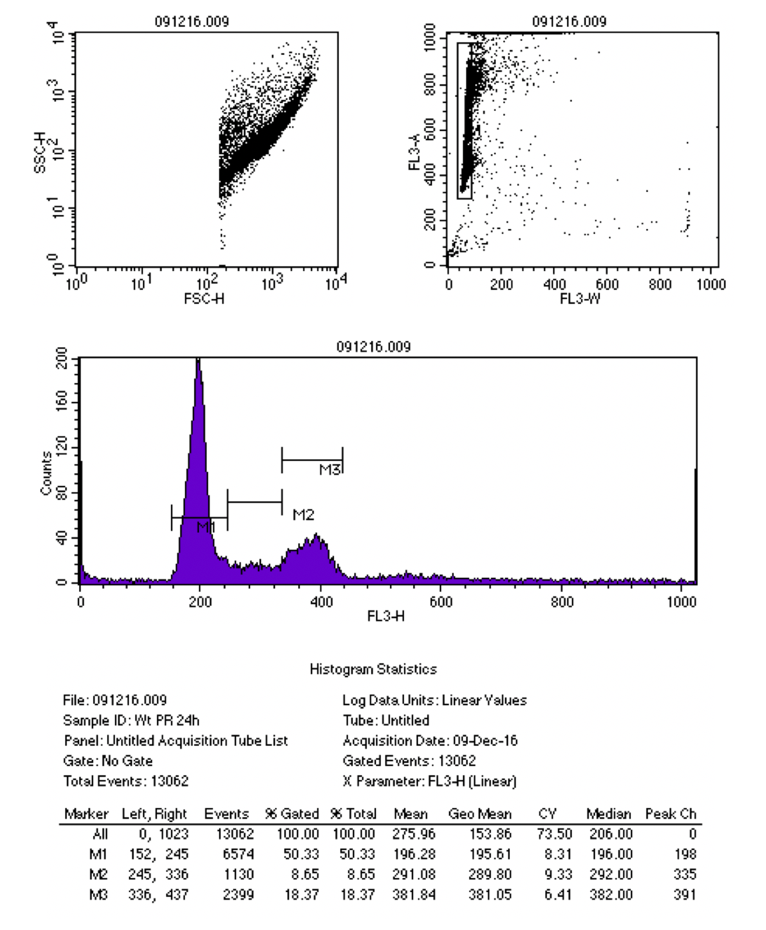


Figure 4—source data 1C. DMEM 24 h


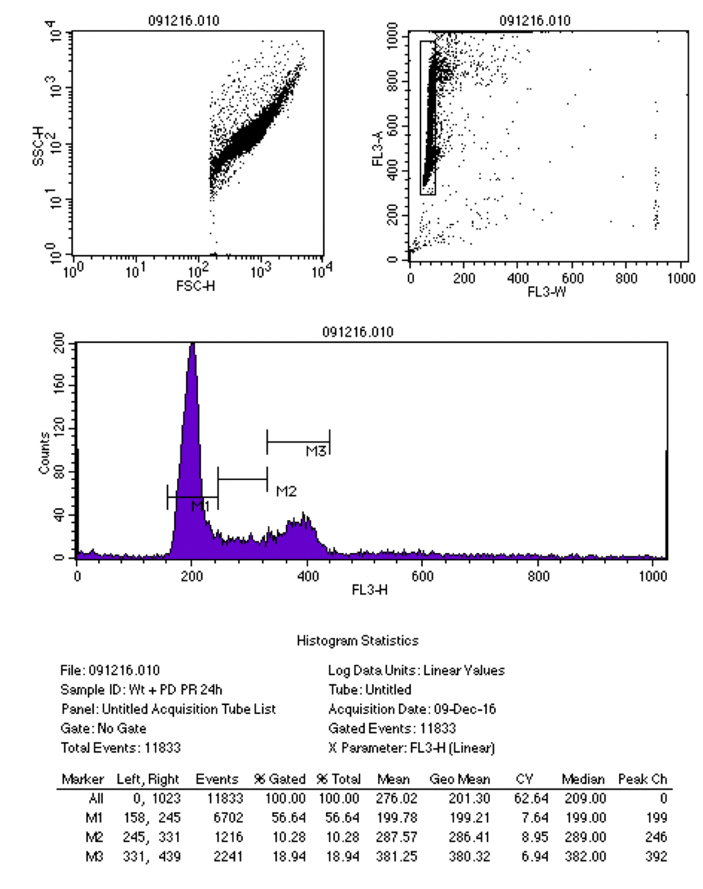


Figure 4—source data 1D. PD0332991 24 h


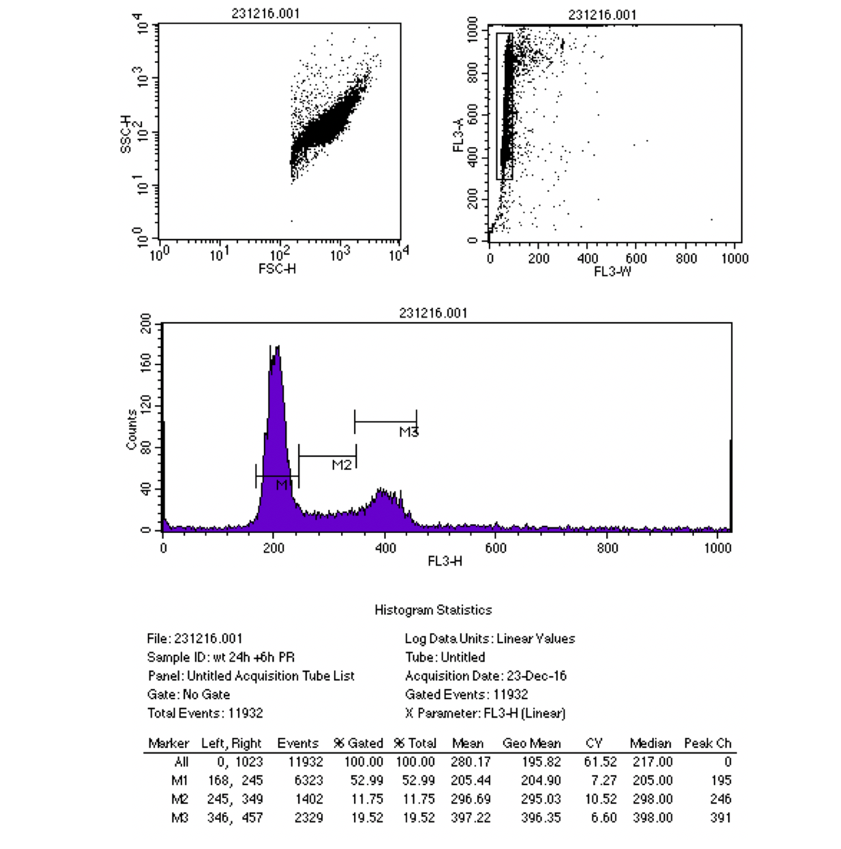


Figure 4—source data 1E. DMEM 6 h


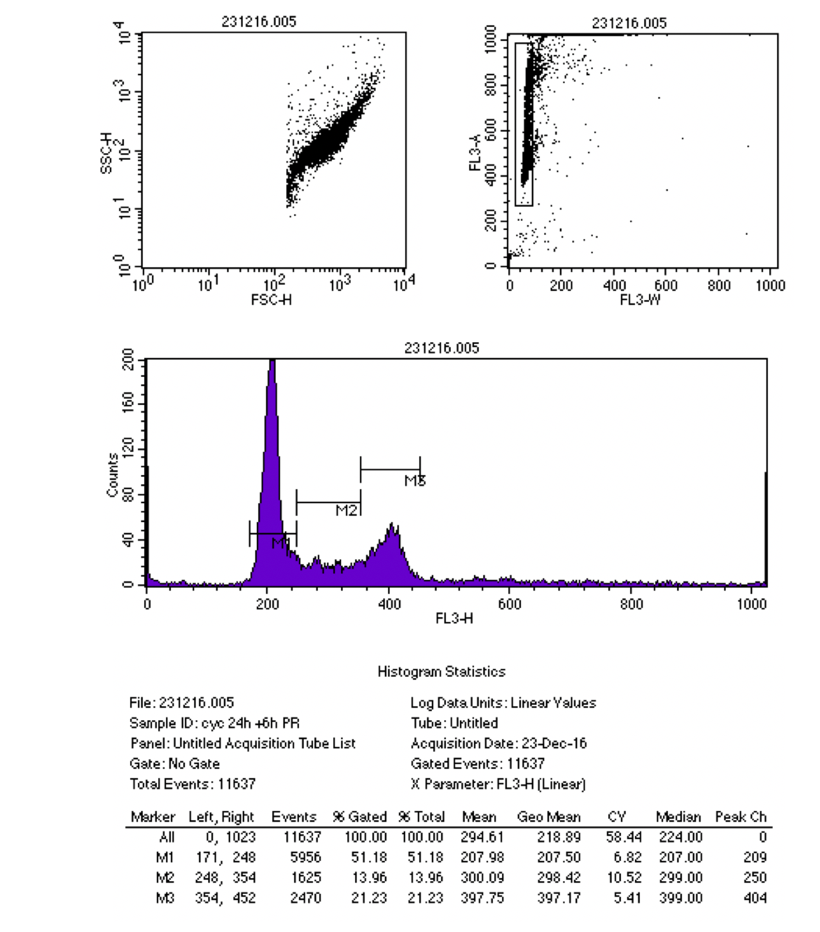


Figure 4—source data 1F. Cyc 6 h


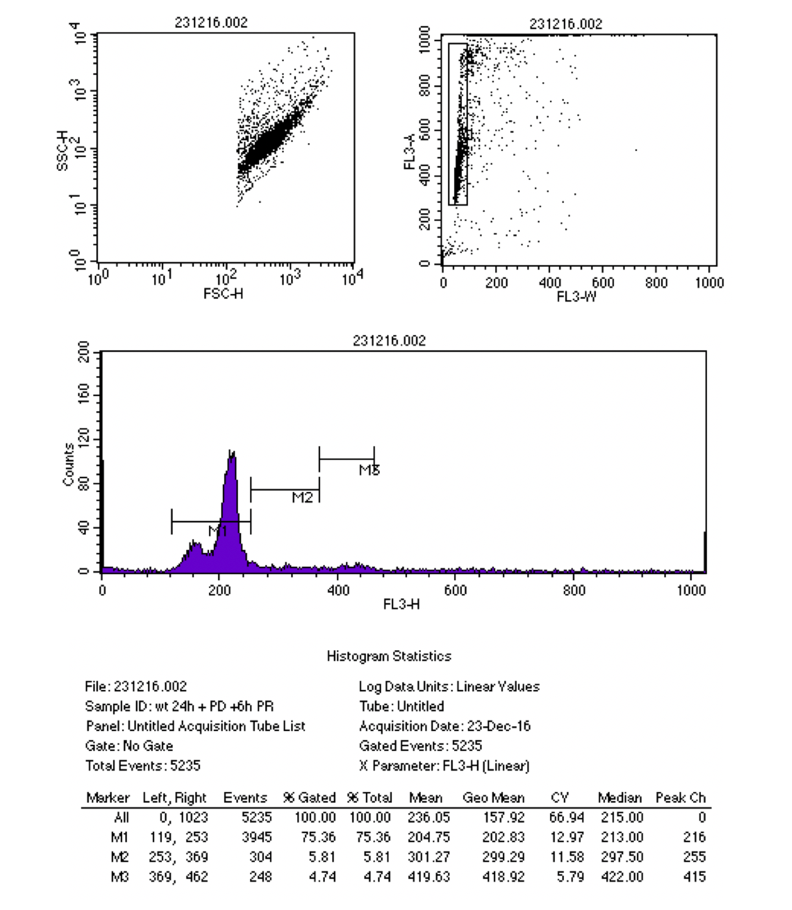


Figure 4—source data 1G. PD0332991 6h


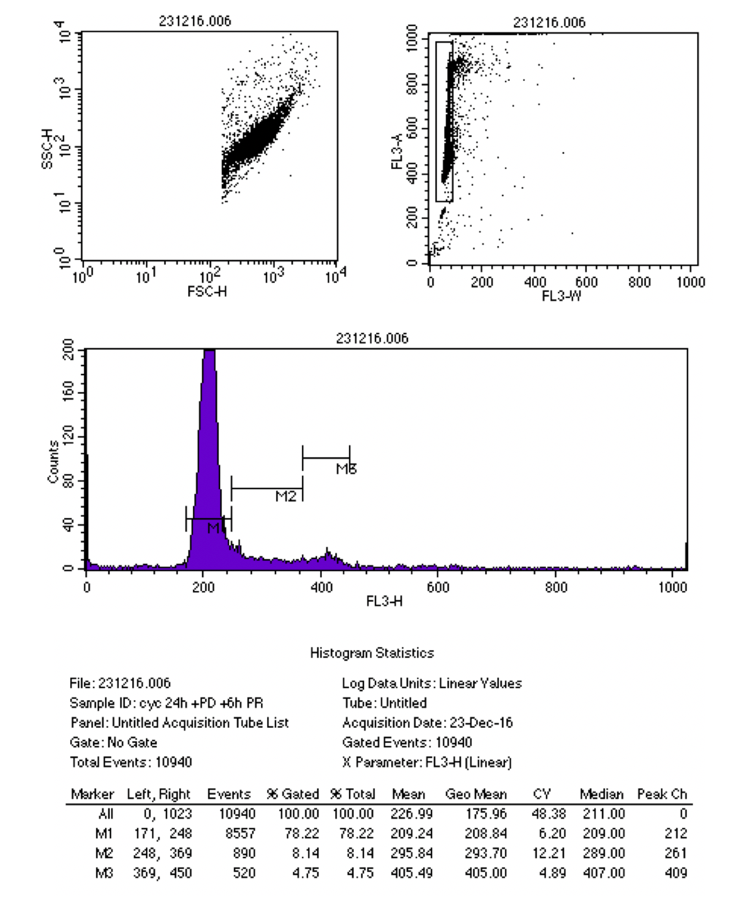


Figure 4—source data 1H. Cyc/PD0332991 6h


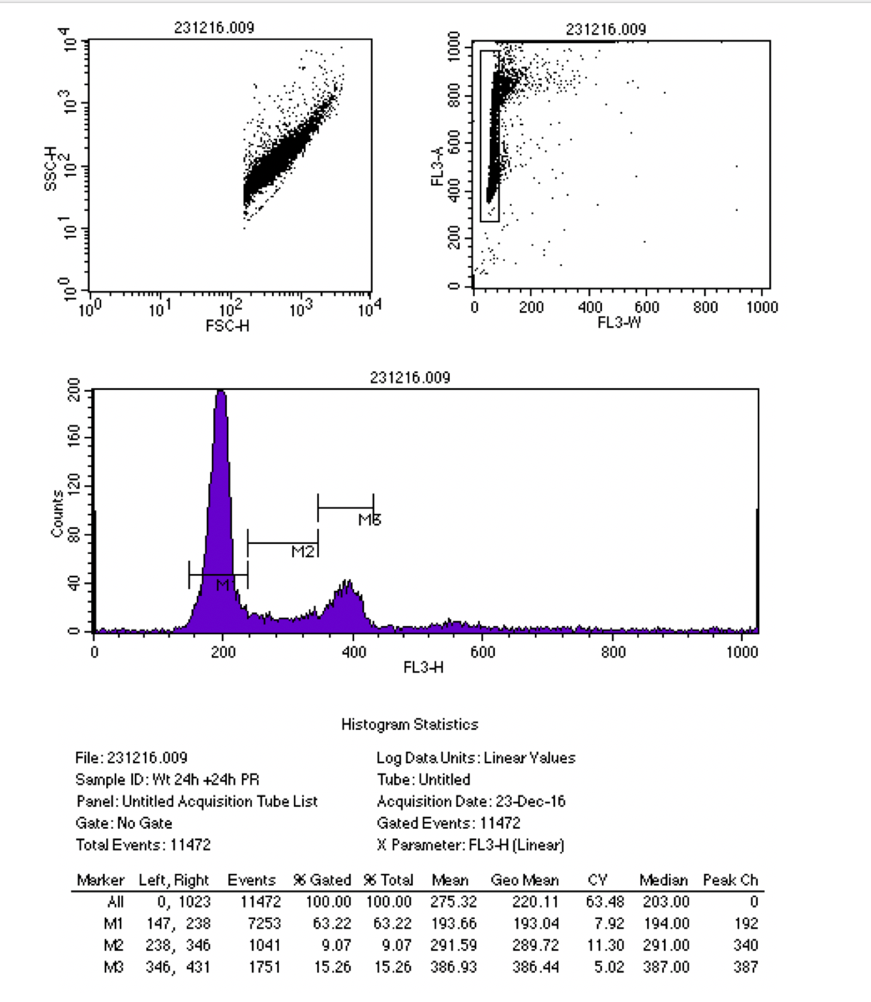


Figure 4—source data 1I. DMEM 24h


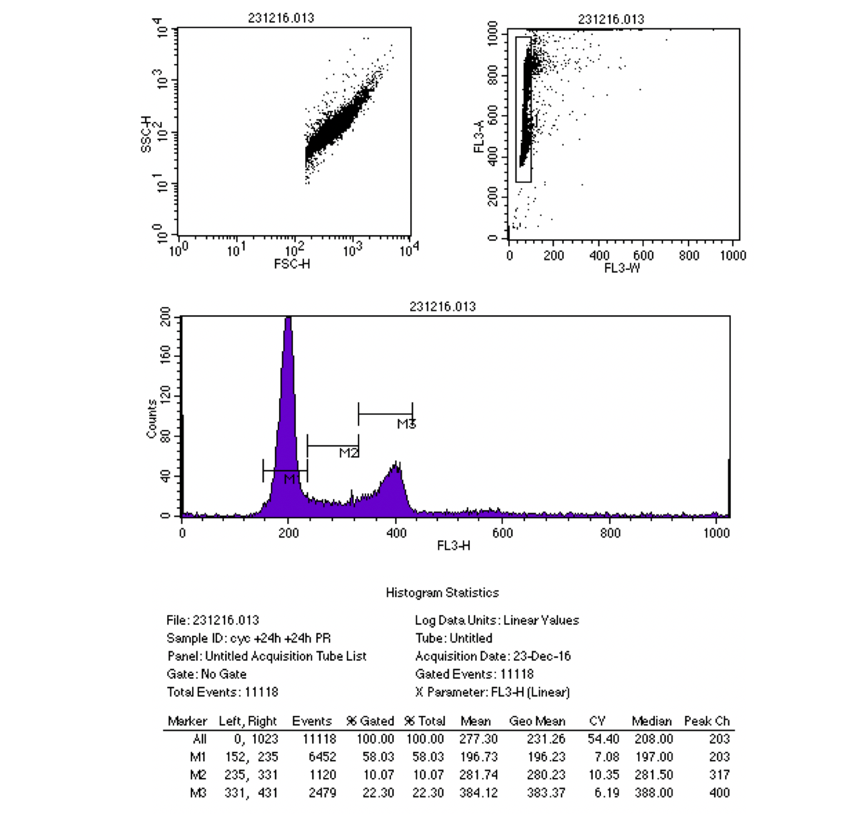


Figure 4—source data 1J. Cyc 24h


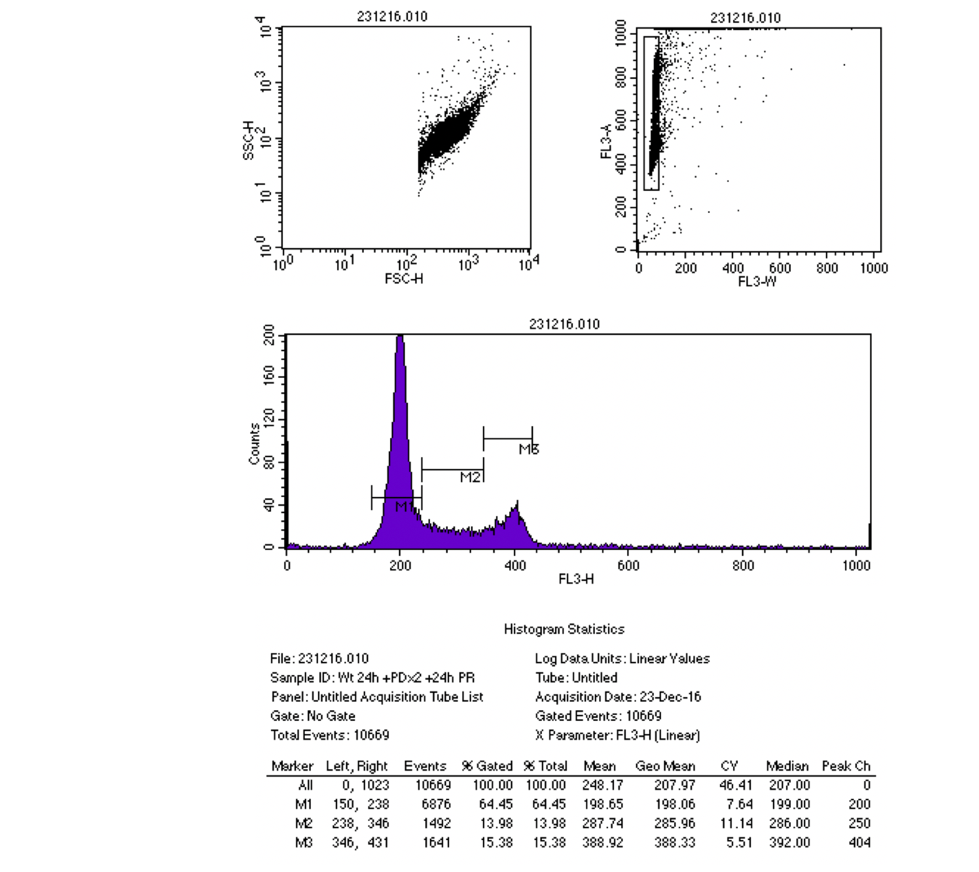


Figure 4—source data 1K. PD0332991 24h


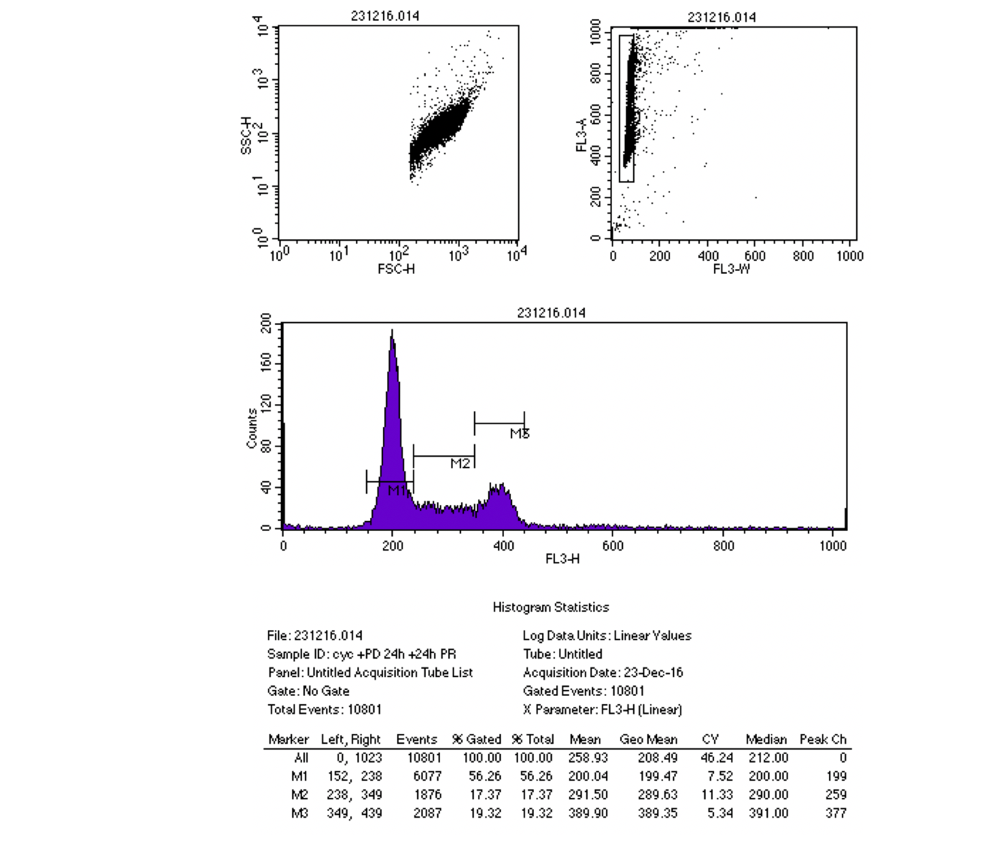


Figure 4—source data 1L. Cyc/PD0 332991 24h
