# Supplementary material for: An autoregulatory cell cycle timer integrates growth and specification in chick wing digit development
Source: eLife. 2019 Sep 23;8:e47625. doi: 10.7554/eLife.47625 (PMC6777937; doi:10.7554/eLife.47625)
Supplement: Figure 6—source data 1. — (A) HBC/PBS 24 h. (B) HBC/Bmp2 24 h. (C) Cyc//PBS 24 h. (D) Cyc//Bmp2 24 h. (E) HBC/PBS 48 h. (F) HBC/Bmp2 48 h. (G) Cyc/PBS 48 h. (H) Cyc/Bmp2 48 h. [file elife-47625-fig6-data1.docx]

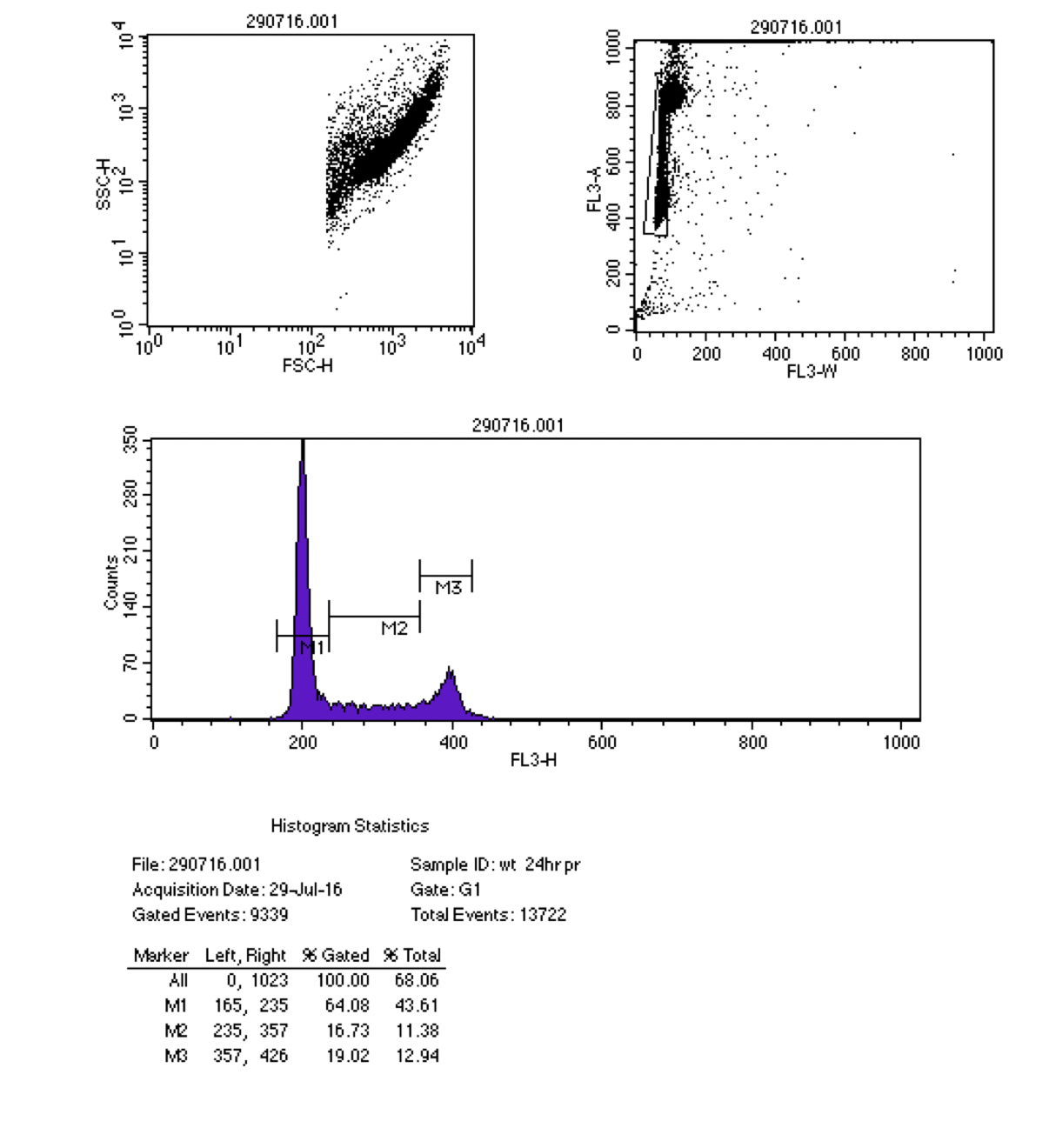


Figure 6—source data 1A. HBC/PBS 24h


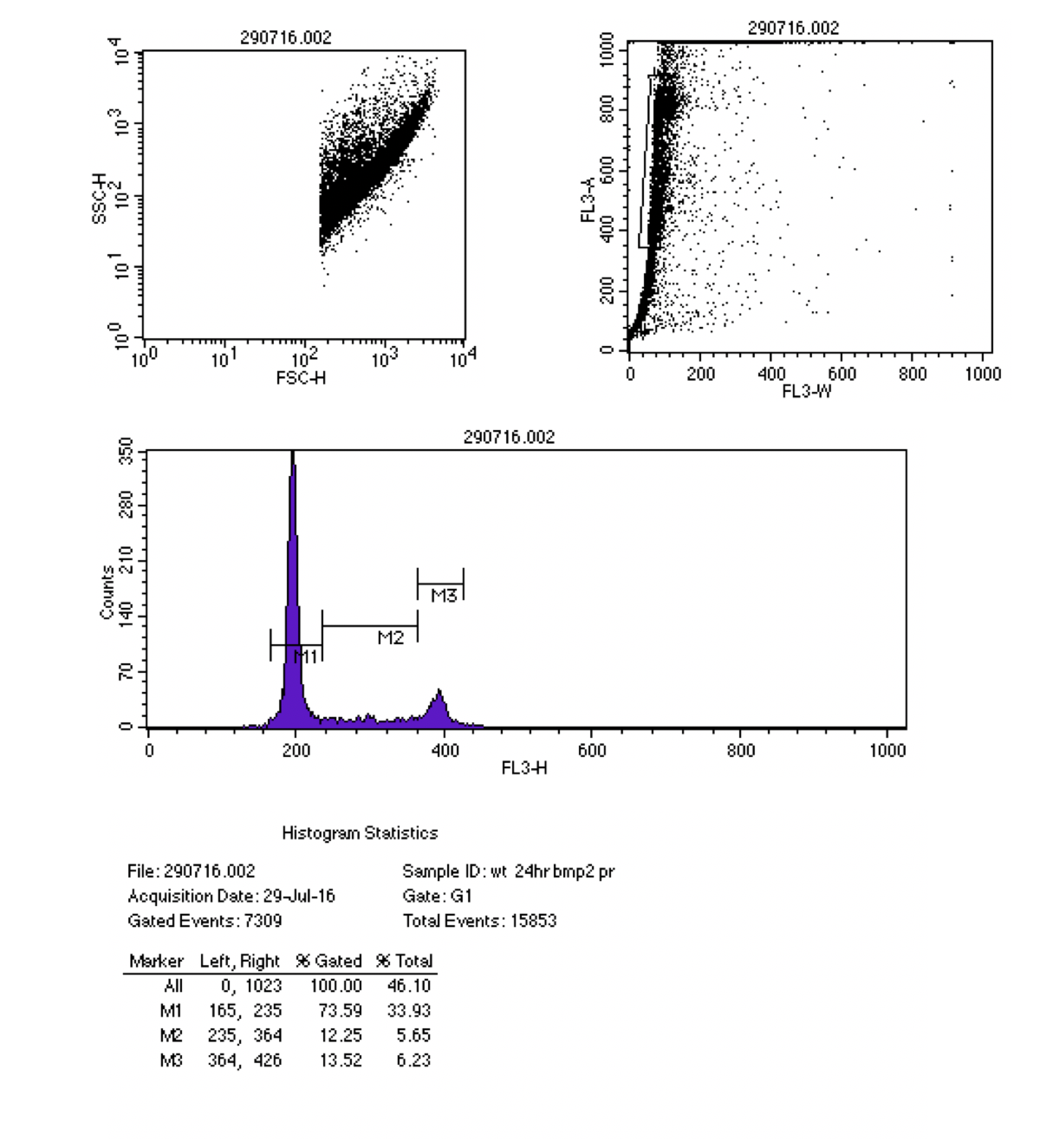


Figure 6—source data 2B. HBC/Bmp2 24h


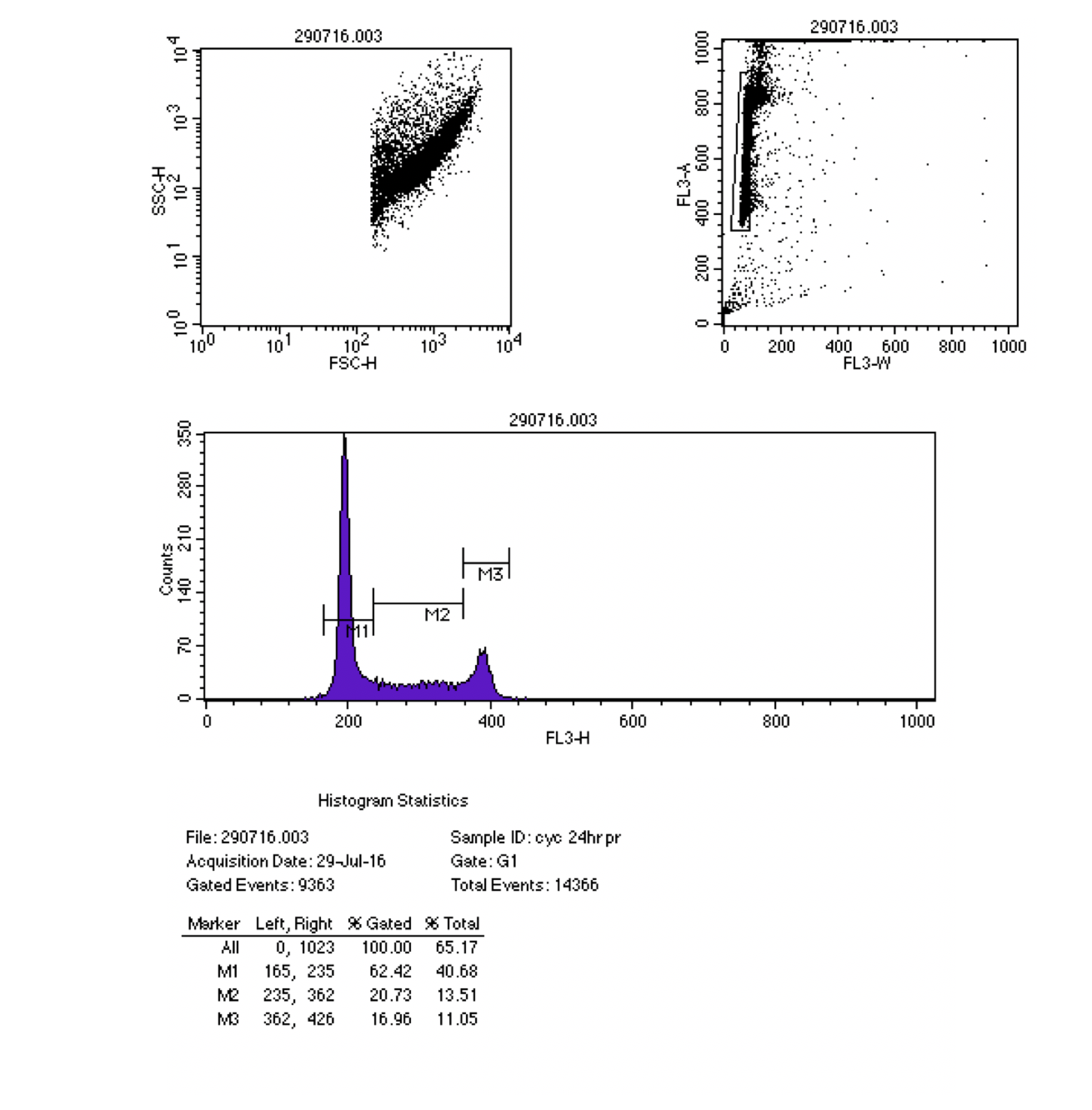


Figure 6—source data 3C. Cyc/PBS 24h


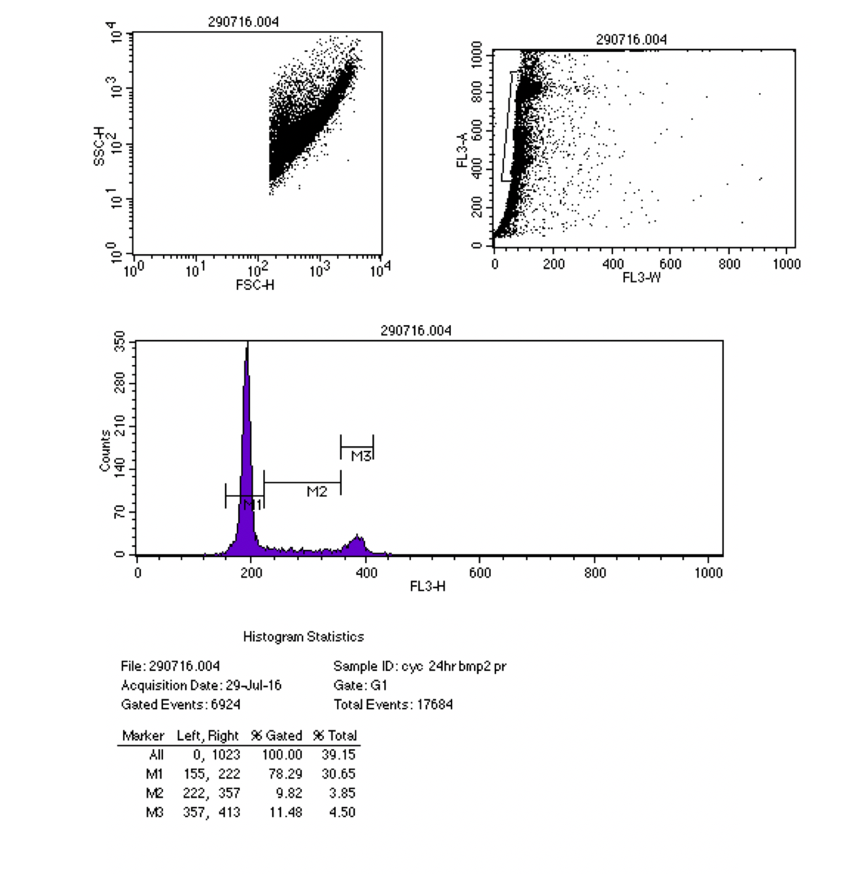


Figure 6—source data 1D. Cyc/Bmp2 24h


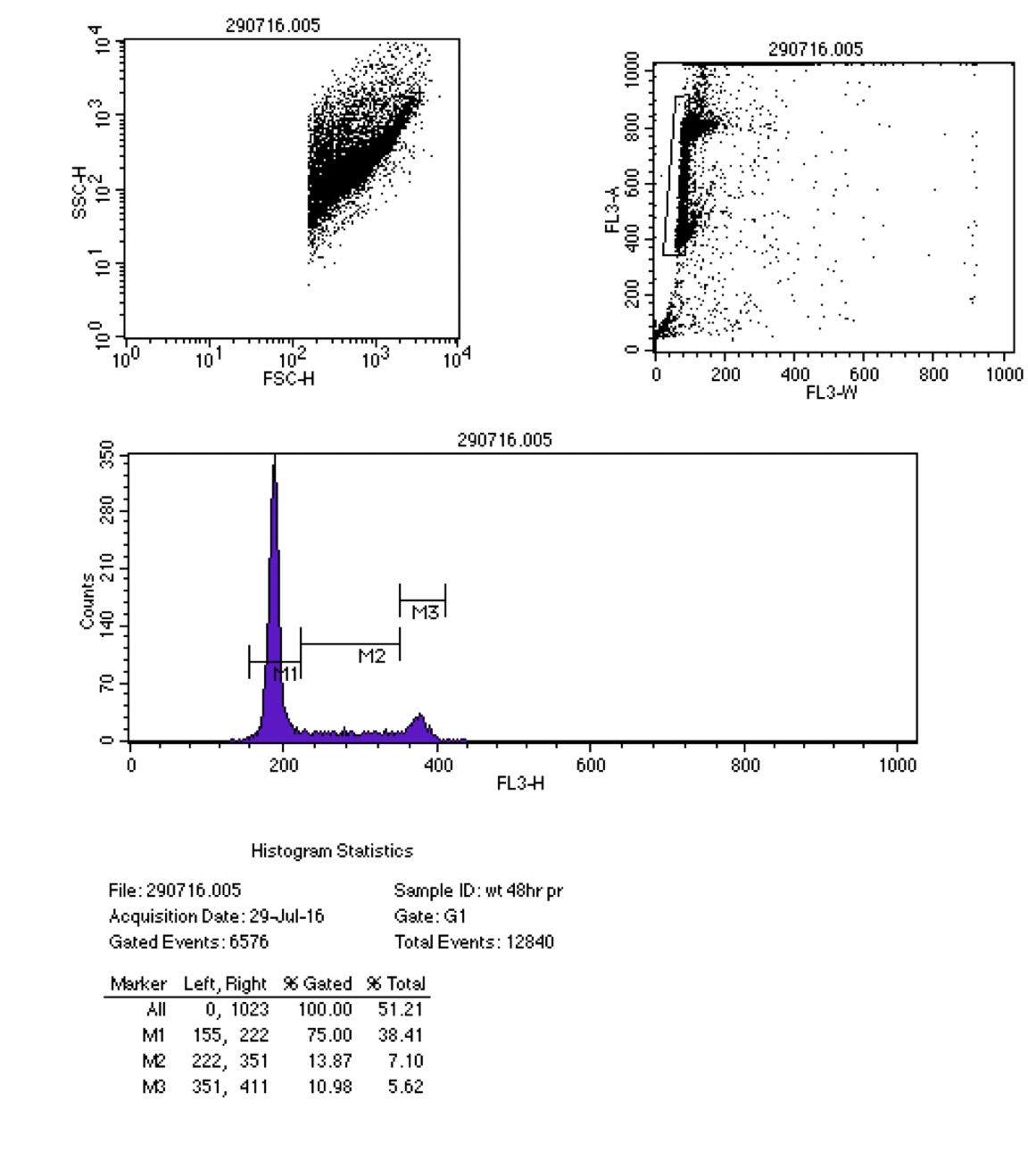


Figure 6—source data 1E. HBC/PBS 48h


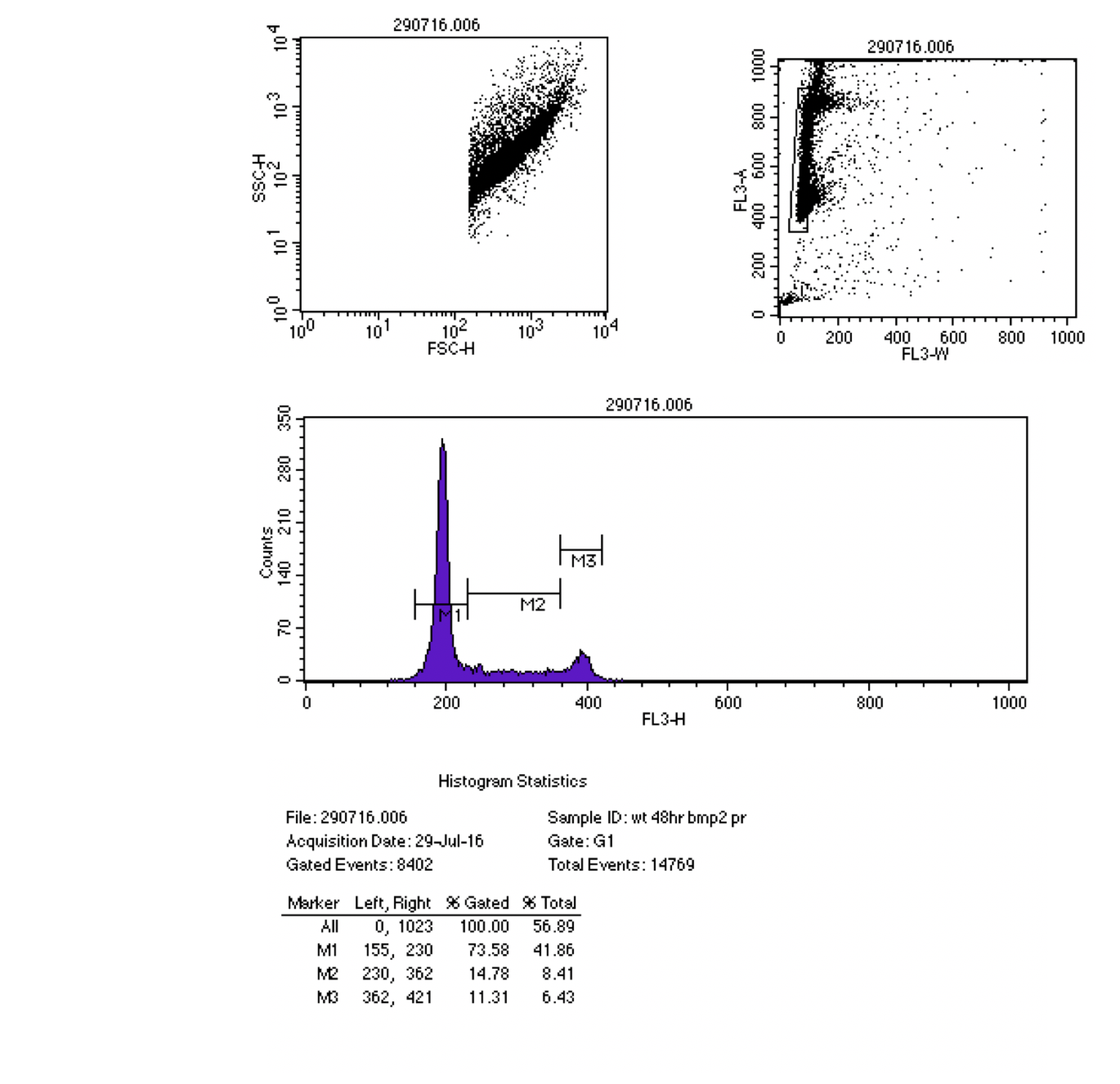


Figure 6—source data 1F. HBC/Bmp2 48 h


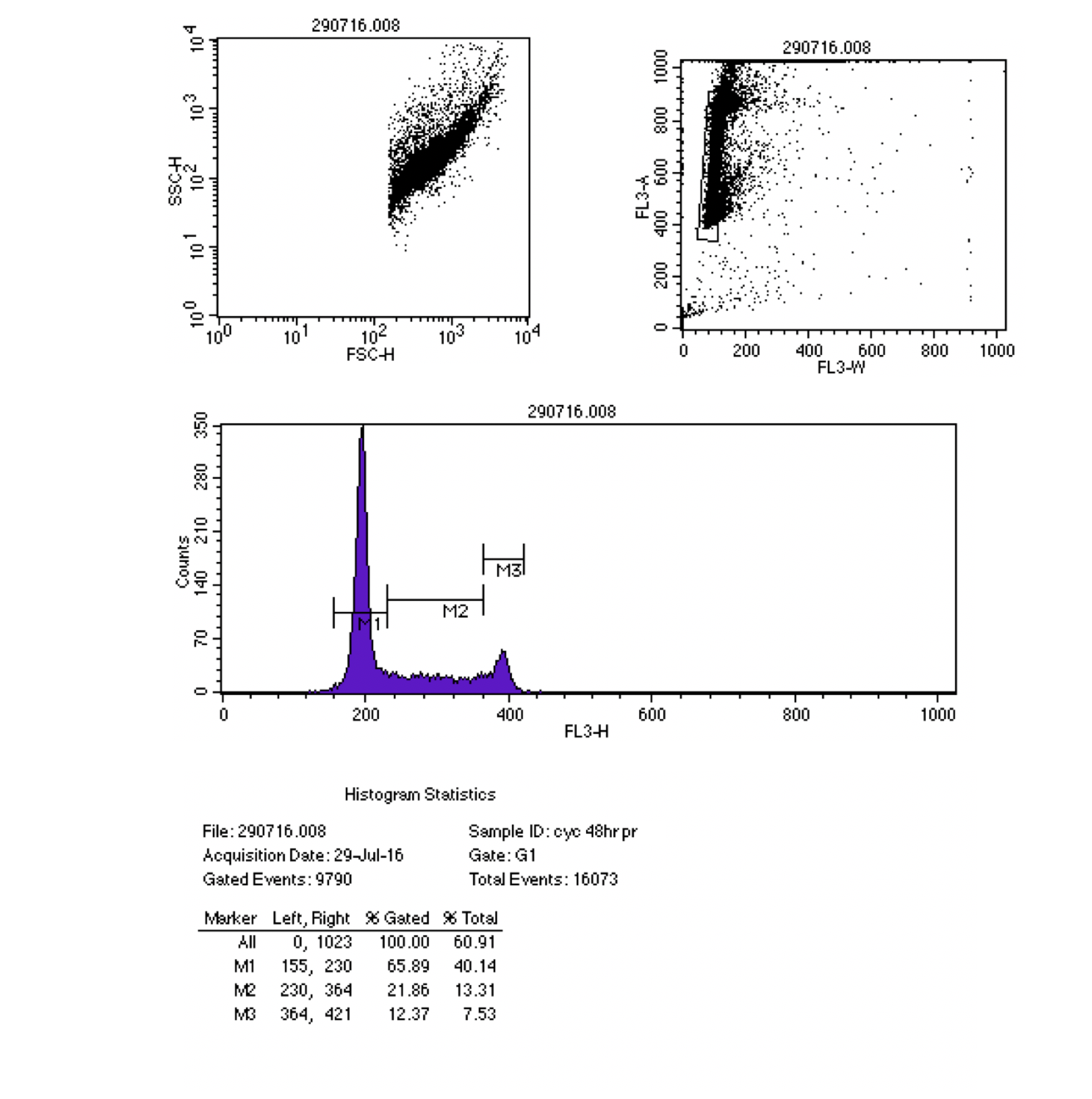


Figure 6—source data 1G. Cyc/PBS 48h


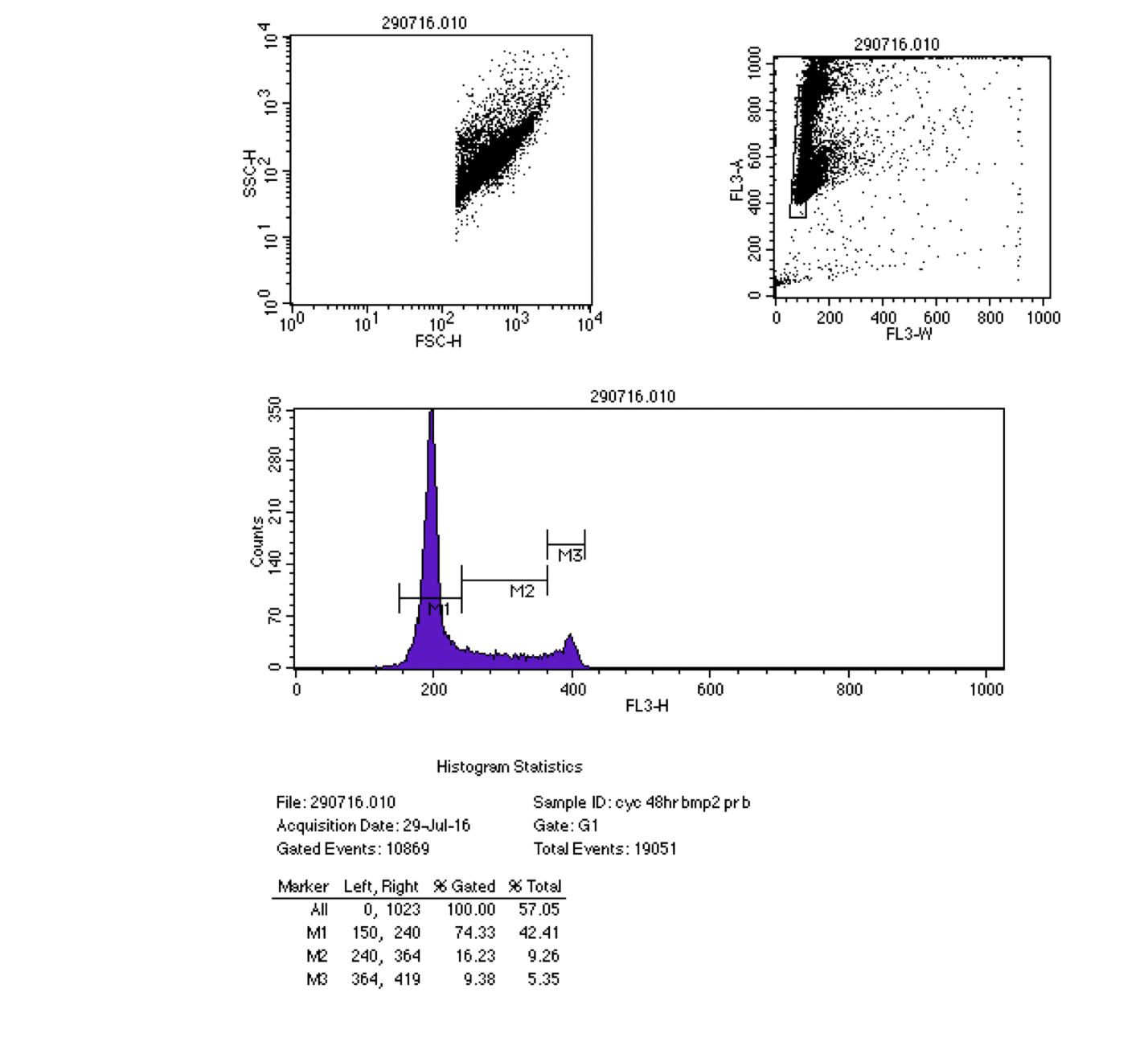


Figure 6—source data 1H. Cyc/Bmp2 48h
